# The influence of surgeon seniority and intestinal failure experience on identifying malnourished patients in emergency general surgery: a national survey

**DOI:** 10.1308/rcsann.2025.0023

**Published:** 2025-06-11

**Authors:** DL Ashmore, TR Wilson, V Halliday, MJ Lee

**Affiliations:** ^1^ University of Sheffield, UK; ^2^ Doncaster and Bassetlaw Teaching Hospitals NHS Foundation Trust, UK; ^3^ University of Birmingham, UK

**Keywords:** Acute care surgery, Malnutrition, Intestinal failure, Nutritional support

## Abstract

**Background:**

Variation exists in how consultant surgeons identify malnutrition in emergency general surgery (EGS) patients. These relate to differences in surgeon knowledge, understanding, ownership and hospital setting. Little is known regarding how these relate to nonconsultant surgeons, or those with experience of intestinal failure (IF).

**Aims:**

This study aimed to characterise the awareness, practice and training of general surgeons in the identification of malnutrition in the emergency setting.

**Methods:**

The survey focused on three domains: perceptions, current practices and nutrition training. Following piloting, EGS surgeons were invited to complete an online survey. Responses were gathered using Qualtrics. Descriptive analysis and associations with surgeon seniority and IF were performed in SPSSv26. Ethical approval was obtained (UREC 050436). Results are reported with reference to the CHERRIES guidelines.

**Results:**

The completion rate was 52.1% (148/284), of whom 49.7% were nonconsultant surgeons and 46.6% had experience of IF. Surgeons from all UK regions completed the survey. There was strong agreement across participants that malnutrition can affect surgical outcomes and identifying it was an important skill for surgeons. However, only 37.2% (55/148) were confident in doing so. Surgeons with IF experience were significantly more confident than those without (49.3% vs 26.6%). Training was reportedly poor, and local teaching or a short course aimed at surgeons in training was considered most helpful in the future.

**Conclusions:**

Identifying malnutrition in EGS is recognised as an important skill most surgeons feel they are lacking. Support for formal training in this area was high.

## Introduction

Many emergency general surgery (EGS) patients are admitted with poor oral intake and are at risk of being malnourished during their admission.^1,2^ It is known that surgical and clinical outcomes are worse in malnourished patients compared with those without malnutrition.^1,3,4^ Recognition of this vulnerable group of patients is paramount to initiate timely nutritional support and potentially improve outcomes.

Studies indicate there is variation in practice in assessing malnutrition in EGS.^5,6^ A lack of training and education for surgeons is considered to be a major barrier to identifying malnourished EGS patients.^5,7^ This has been explored at consultant level, but no studies have looked at views of nonconsultant surgeons, including surgical trainees and associate specialists. One study involving gastroenterology medical trainees has shown low confidence in recognising malnutrition and a lack of training in this area.^8^

Patients with intestinal failure (IF) are complex and require nutritional support. Their care is often managed by specialised centres, or IF units. Surgeons working at these units may develop skills they carry forward in their practice at other centres. However, the influence of a surgeon's IF experience on how malnourished patients are identified has also not been explored.

## Aims

This study explored the perceptions and practice of general surgeons in identifying and managing malnourished EGS patients. The training surgeons had received, and how this could be improved, was also investigated. Secondary aims explored whether differences exist according to surgeon seniority or experience of IF.

## Methods

A cross-sectional survey was designed with reference to current nutrition guidelines and the existing literature.^9–12^ The results from the survey are reported with reference to the CHERRIES guidelines.^13^

### Study design

The study was designed in two sections that were delivered simultaneously to the same population to limit burden to participants. This included the survey as described here, as well as a discrete choice experiment (DCE). In summary, a DCE can be helpful to understand decision making regarding treatment choices, particularly where there is uncertainty of best practice or individual preference is significant in the decision-making process. Important variables involved in decision making were used to randomly generate hypothetical scenarios.^14–17^ Participants were then asked whether they would start nutritional support in each scenario, allowing investigators to derive the relevance of each variable in the decision-making process. The DCE will be reported in due course as the underlying aims, methodology and findings were markedly different.

The relevant questions pertaining to the survey reported here are provided (Appendix 1).

This survey captured limited demographic data about respondents including grade of surgeon, self-reported experience of managing patients with IF and current region of work. There were three main domains:

• Perceptions around malnutrition in EGS
• Current practices in identifying malnutrition
• Nutrition training and the future

### Ethical approval and informed consent

University of Sheffield ethical approval was obtained (UREC 050436). Potential participants were invited by email to read a participant information sheet. This explained the purpose of the study, how data would be stored, the study investigators and the duration of the survey. Survey consent was implied by participants clicking on a link in the invitation email, and subsequently on another link agreeing to continue on the welcome page.

### Recruitment

Surgeons working at registrar level or above were eligible to complete the survey. This included surgeons in training and fellows postcertificate of completion of training, as well as SAS (staff grade, associate specialist and specialty doctors) and trust-grade surgeons. This wide surgical cohort was labelled ‘nonconsultants’ during analysis and will be referred to as such throughout.

There were 4,889 general surgeons in the NHS in March 2022, some of whom will be breast surgeons and do not participant on an EGS rota.^18^ Given the target population is relatively small, calculating a minimum sample size was considered unhelpful, and a focus on the widest possible recruitment was implemented instead.

### Development and pretesting

The survey was piloted among five consultant and nonconsultant general surgeons, assessing for face and content validity using a feedback form based on the validated QQ-10 tool.^19^ Additional feedback regarding survey design, question wording and completion time was discussed among the expert panel.

Responses from the pilot study confirmed the survey was succinct, easy to complete and relevant. Free text responses advised the need for fewer questions per page, and this was incorporated into the final survey design. The mean time for completion was five minutes for the survey section of the study.

### Survey administration

The final study was publicised to general surgeons by several routes. This included: surgeons who had previously registered an interest in studies undertaken by the research team; to members of the Association of Surgeons of Great Britain and Ireland (ASGBI), for which the study had received research support and via social media platforms to surgical trainee- and EGS-specific groups. Responses were gathered using Qualtrics survey software.

The study opened in December 2023 for ten weeks with two additional reminders to complete it. There were no incentives. Responses were anonymous, although participants were able to leave their email if they wished to be contacted in the future, be informed of findings or avoid reminder emails. Questions were from a drop-down list, a five-point Likert question-matrix with a ‘do not know’ option, or a selection of options from a menu. Instructions to ‘select one’ or ‘select all that apply’ were given as appropriate. Questions were formatted adaptively with free text boxes also offered where appropriate. There were up to eight questions per page displayed on seven pages including the opening and closure pages; a progress bar was visible. Mandatory questions were indicated, and participants were able to review answers until submission.

### Analysis

Descriptive analyses were performed in IBM SPSSv26. Associations of response medians with surgeon seniority and experience of IF were performed using appropriate tests of significance, namely Mann–Whitney *U*. Representative statements from free text data are presented in the findings.

## Results

### Survey results

A total of 284 responses were recorded, of which 149 participants completed the survey. One response was excluded as the participant was a core surgical trainee and not eligible to participate. This resulted in an overall completion rate of 52.1% (148/284). There was an approximately even number of consultants and nonconsultants (50.7% vs 49.3%), and surgeons with experience of IF (46.6%, 69/148) and those without (53.4%, 79/148). Half of the nonconsultant surgeons were trainee registrars (50.1%, 37/73). Ten consultants (13.3%, 10/75) worked in an IF unit. At least one participant from all training regions in the UK completed the survey. A summary of the included participants is shown (Table 1).

**Table 1:** Summary characteristics of the included participants

Scenario	Responses, % (*n*)
Grade	
Consultant	50.7 (75)
Nonconsultant	49.3 (73)
ST7-8/post-CCT fellow	14.9 (22)
ST3-6	25.0 (37)
SAS doctor, associate specialist, trust grade	9.5 (14)
Consultants with IF experience	
Yes	57.3 (43)
No	42.7 (32)
Participants with IF experience	
Yes	46.6 (69)
No	53.4 (79)
Consultants working in an IFU	
Yes	13.3 (10)
No	86.7 (65)
Region of work	
Yorkshire and the Humber	43.2 (64)
North West (North West)	9.5 (14)
West Midlands	7.4 (11)
Kent, Surrey and Sussex	4.1 (6)
South West (Severn)	3.4 (5)
Wessex	3.4 (5)
Thames Valley	2.7 (4)
North West (Mersey)	2.7 (4)
Wales	2.7 (4)
South West (Peninsula)	2.7 (4)
North East	2.0 (3)
North West London	2.0 (3)
Northern Ireland	2.0 (3)
East of England	2.0 (3)
Ireland	2.0 (3)
Scotland (South East – Edinburgh)	1.4 (2)
East Midlands (South)	1.4 (2)
East Midlands (North)	1.4 (2)
Scotland (West – Glasgow)	0.7 (1)
South London (South West)	0.7 (1)
North Central and East London	0.7 (1)
South London (South East)	0.7 (1)
Scotland (North – Aberdeen)	0.7 (1)
Scotland (East – Dundee)	0.7 (1)

CCT = certificate of completion of training; IF = intestinal failure; IFU = intestinal failure unit; SAS = specialty and associate specialist ; ST = specialty trainee.

### Perceptions around malnutrition in EGS

**Figure 1:**
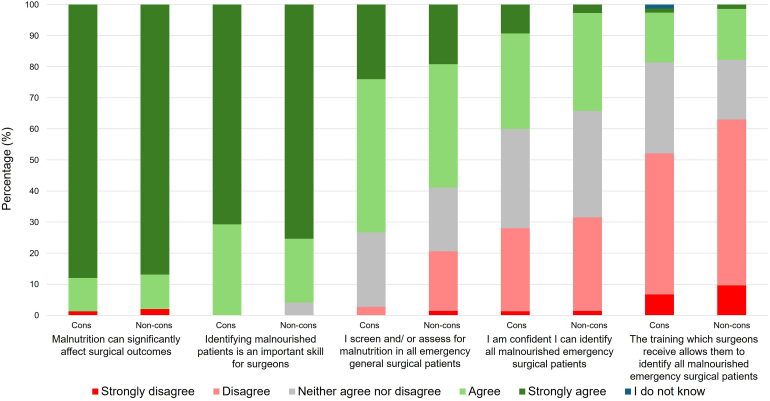
Perceptions of consultant and nonconsultant surgeons. cons = consultant; non-cons = nonconsultant.

Regardless of experience of IF, almost all consultant and nonconsultant surgeons agreed or strongly agreed that malnutrition can significantly affect surgical outcomes (98.0%, 145/148) and identifying malnourished patients was an important skill for surgeons (98.0%, 145/148) (Table 2, Figure 1).

**Table 2:** Awareness, practice, responsibility and organisational support according to surgeon seniority and experience of intestinal failure

Question	Role	*n*	Strongly disagree	Disagree	Neither agree nor disagree	Agree	Strongly agree	I do not know
Malnutrition can significantly affect surgical outcomes	Cons	75	1.3 (1)	0.0 (0)	0.0 (0)	10.7 (8)	88.0 (66)	0.0 (0)
	Non-cons	73	3.2 (2)	0.0 (0)	0.0 (0)	11.0 (8)	86.3 (63)	0.0 (0)
	IF experience	69	2.9 (2)	0.0 (0)	0.0 (0)	5.8 (4)	91.3 (63)	0.0 (0)
	No IF experience	79	1.3 (1)	0.0 (0)	0.0 (0)	15.2 (12)	83.5 (66)	0.0 (0)
	Total	148	2.0 (3)	0.0 (0)	0.0 (0)	10.8 (16)	87.2 (129)	0.0 (0)
Identifying malnourished patients is an important skill for surgeons	Cons	75	0.0 (0)	0.0 (0)	0.0 (0)	29.3 (22)	70.7 (53)	0.0 (0)
	Non-cons	73	0.0 (0)	0.0 (0)	4.1 (3)	20.5 (15)	75.3 (55)	0.0 (0)
	IF experience	69	0.0 (0)	0.0 (0)	0.0 (0)	23.2 (16)	76.8 (53)	0.0 (0)
	No IF experience	79	0.0 (0)	0.0 (0)	3.8 (3)	26.6 (21)	69.6 (55)	0.0 (0)
	Total	148	0.0 (0)	0.0 (0)	2.0 (3)	25.0 (37)	73.0 (108)	0.0 (0)
I screen and/ or assess for malnutrition in all emergency general surgical patients	Cons	75	0.0 (0)	2.7 (2)	24.0 (18)	49.3 (37)	24.0 (18)	0.0 (0)
	Non-cons	73	1.4 (1)	19.2 (14)	20.5 (15)	39.7 (29)	19.2 (14)	0.0 (0)
	IF experience	69	1.4 (1)	8.7 (6)	17.4 (12)	43.5 (30)	29.0 (20)	0.0 (0)
	No IF experience	79	0.0 (0)	12.7 (10)	26.6 (21)	45.6 (36)	15.2 (12)	0.0 (0)
	Total	148	0.7 (1)	10.8 (16)	22.3 (33)	44.6 (66)	21.6 (32)	0.0 (0)
I am confident I can identify all malnourished emergency surgical patients	Cons	75	1.3 (1)	26.7 (20)	32.0 (24)	30.7 (23)	9.3 (7)	0.0 (0)
	Non-cons	73	1.4 (1)	30.1 (22)	34.2 (25)	31.5 (23)	2.7 (2)	0.0 (0)
	IF experience	69	1.4 (1)	23.2 (16)	26.1 (18)	37.7 (26)	11.6 (8)	0.0 (0)
	No IF experience	79	1.3 (1)	32.9 (26)	39.2 (31)	25.3 (20)	1.3 (1)	0.0 (0)
	Total	148	1.4 (2)	28.4 (42)	33.1 (49)	31.1 (46)	6.1 (9)	0.0 (0)
Surgeons are primarily responsible for identifying malnutrition in emergency surgical patients	Cons	75	0.0 (0)	9.3 (7)	26.7 (20)	50.7 (38)	13.3 (10)	0.0 (0)
	Non-cons	73	0.0 (0)	9.6 (7)	24.7 (18)	46.6 (34)	19.2 (14)	0.0 (0)
	IF experience	69	0.0 (0)	7.2 (5)	30.4 (21)	47.8 (33)	14.5 (10)	0.0 (0)
	No IF experience	79	0.0 (0)	11.4 (9)	21.5 (17)	49.4 (39)	17.7 (14)	0.0 (0)
	Total	148	0.0 (0)	9.5 (14)	25.7 (38)	48.6 (72)	16.2 (24)	0.0 (0)
Surgeons are primarily responsible for managing malnutrition in emergency surgical patients	Cons	75	1.3 (1)	14.7 (11)	29.3 (22)	36.0 (27)	18.7 (14)	0.0 (0)
	Non-cons	73	1.4 (1)	12.3 (9)	26.0 (19)	47.9 (35)	12.3 (9)	0.0 (0)
	IF experience	69	2.9 (2)	10.1 (7)	21.7 (15)	46.4 (32)	18.8 (13)	0.0 (0)
	No IF experience	79	0.0 (0)	16.5 (13)	32.9 (26)	38.0 (30)	12.7 (10)	0.0 (0)
	Total	148	1.4 (2)	13.5 (20)	27.7 (41)	41.9 (62)	15.5 (23)	0.0 (0)
My organisation provides the support I need to identify and manage all malnourished emergency surgical patients	Cons	75	0.0 (0)	12.0 (9)	30.7 (23)	36.0 (27)	20.0 (15)	1.3 (1)
	Non-cons	73	1.4 (1)	23.3 (17)	27.4 (20)	39.7 (29)	6.8 (5)	1.4 (1)
	IF experience	69	0.0 (0)	14.5 (10)	29.0 (20)	36.2 (25)	18.8 (13)	1.4 (1)
	No IF experience	79	1.3 (1)	20.3 (16)	29.1 (23)	39.2 (31)	8.9 (7)	1.3 (1)
	Total	148	0.7 (1)	17.6 (26)	29.1 (43)	37.8 (56)	13.5 (20)	1.4 (2)

Responses as % (*n*). Note ST7-8 category also includes post-CCT fellows.

CCT = certificate of completion of training; cons = consultant; IF = intestinal failure; IFU = intestinal failure unit; non-cons = nonconsultant; SAS = specialty and associate specialist; ST = specialty trainee.

Overall, two-thirds of respondents regularly screened for malnutrition (66.2%, 98/148; Table 2). Surgeons with IF experience did this significantly more than colleagues without IF experience (72.5% vs 60.8%, *n*=148, *p*=0.050; Table 2 and Supplementary Table S1). The screening rates between consultant surgeons and nonconsultant surgeons were also significantly different (74.7% vs 58.9%, *n*=148, *p*=0.035) (Supplementary Table S2). The commonest reasons reported for not screening all the time were it would not change immediate management, a lack of appropriate training and a lack of time (Supplementary Table S3). These findings were reflected across surgeon grade and IF experience.

In total, only 37.2% (55/148) of all surgeons were confident they could identify malnutrition in EGS patients (Table 2, Figure 1). Confidence among surgeons was low across all groups. Although surgeons with IF experience were significantly more confident compared with surgeons with no IF experience (49.3% vs 26.6%, *n*=148, *p*=0.008; Table 2 and Supplementary Table S1), still more than half of these experts lacked confidence.

Overall, surgeons felt more responsible for identifying malnourished patients (64.9%, 96/148) rather than managing their malnutrition (57.4%, 85/148) (Table 2). This difference is greater in consultant surgeons than in nonconsultant surgeons. Surgeons with IF experience felt primarily responsible for managing malnourished patients more than those without IF experience at both consultant and nonconsultant grade. However, none of these findings were significant (Supplementary Tables S1 and S2). Where surgeons indicated they were not responsible for identifying nor managing malnutrition, it was felt that these roles fell predominantly to dietitians and the nutrition support team, respectively (Supplementary Tables S4 and S5).

Only half (51.3%, 76/148) of surgeons felt their organisation supported them to identify and manage malnutrition in EGS (Table 2). Although consultant surgeons were significantly more likely than nonconsultants to say there was adequate support (*n*=148, consultant median response=4, nonconsultant median response=3, *p*=0.035) (Supplementary Table S2), over 40% were indifferent or disagreed. In general, surgeons with IF experience were more favourable that organisational support was available than surgeons without IF experience (Table 2).

### Current practices in identifying malnutrition

**Figure 2:**
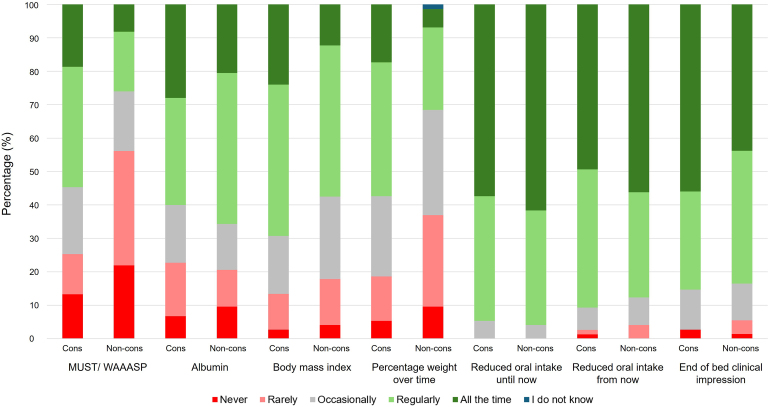
Methods to identify malnutrition by consultant and nonconsultant surgeons. cons = consultant; MUST = Malnutrition Universal Screening Tool; non-cons = nonconsultant; WAASP = weight, appetite, ability to eat, stress factor, pressure ulcer/wound.

Overall, 95.3%, 89.2% and 84.6% of surgeons used ‘reduced oral intake until now’, ‘reduced oral intake from now’ and an end of the bed clinical impression to identify malnutrition, respectively (Table 3, Figure 2).

**Table 3:** Measures used to determine nutritional status according to surgeon seniority and experience of intestinal failure

Question	Role	*n*	Never	Rarely	Occasionally	Regularly	All the time	I do not know
MUST or WAASP	Cons	75	13.3 (10)	12.0 (9)	20.0 (15)	36.0 (27)	18.7 (14)	0.0 (0)
	Non-cons	73	21.9 (16)	34.2 (25)	17.8 (13)	17.8 (13)	8.2 (6)	0.0 (0)
	IF experience	69	10.1 (7)	15.9 (11)	15.9 (11)	34.8 (24)	23.2 (16)	0.0 (0)
	No IF experience	79	24.1 (19)	29.1 (23)	21.5 (17)	20.3 (16)	5.1 (4)	0.0 (0)
	Total	148	17.6 (26)	23.0 (34)	18.9 (28)	27.0 (40)	13.5 (20)	0.0 (0)
Albumin	Cons	75	6.7 (5)	16.0 (12)	17.3 (13)	32.0 (24)	28.0 (21)	0.0 (0)
	Non-con	73	9.6 (7)	11.0 (8)	13.7 (10)	45.2 (33)	20.5 (15)	0.0 (0)
	IF experience	69	5.8 (4)	18.8 (13)	14.5 (10)	36.2 (25)	24.6 (17)	0.0 (0)
	No IF experience	79	10.1 (8)	8.9 (7)	16.5 (13)	40.5 (32)	24.1 (19)	0.0 (0)
	Total	148	8.1 (12)	13.5 (20)	15.5 (23)	38.5 (57)	24.3 (36)	0.0 (0)
Body mass index	Cons	75	2.7 (2)	10.7 (8)	17.3 (13)	45.3 (34)	24.0 (18)	0.0 (0)
	Non-cons	73	4.1 (3)	13.7 (10)	24.7 (18)	45.2 (33)	12.3 (9)	0.0 (0)
	IF experience	69	4.3 (3)	10.1 (7)	23.2 (16)	46.4 (32)	15.9 (11)	0.0 (0)
	No IF experience	79	2.5 (2)	13.9 (11)	19.0 (15)	44.3 (35)	20.3 (16)	0.0 (0)
	Total	148	3.4 (5)	12.2 (18)	20.9 (31)	45.3 (67)	18.2 (27)	0.0 (0)
Percentage weight loss over time	Cons	75	5.3 (4)	13.3 (10)	24.0 (18)	40.0 (30)	17.3 (13)	0.0 (0)
	Non-cons	73	9.6 (7)	27.4 (20)	31.5 (23)	24.7 (18)	5.5 (4)	1.4 (1)
	IF experience	69	5.8 (4)	10.1 (7)	29.0 (20)	39.1 (27)	15.9 (11)	0.0 (0)
	No IF experience	79	8.9 (7)	29.1 (23)	27.8 (22)	26.6 (21)	7.6 (6)	0.0 (0)
	Total	148	7.4 (11)	20.3 (30)	27.7 (41)	32.4 (48)	11.5 (17)	0.7 (1)
Reduced oral intake until now	Cons	75	0.0 (0)	0.0 (0)	5.3 (4)	37.3 (28)	57.3 (43)	0.0 (0)
	Non-cons	73	0.0 (0)	0.0 (0)	4.1 (3)	34.2 (25)	61.6 (45)	0.0 (0)
	IF experience	69	0.0 (0)	0.0 (0)	7.2 (5)	34.8 (24)	58.0 (40)	0.0 (0)
	No IF experience	79	0.0 (0)	0.0 (0)	2.5 (2)	36.7 (29)	60.8 (48)	0.0 (0)
	Total	148	0.0 (0)	0.0 (0)	4.7 (7)	35.8 (53)	59.5 (88)	0.0 (0)
Reduced oral intake from now	Cons	75	1.3 (1)	1.3 (1)	6.7 (5)	41.3 (31)	49.3 (37)	0.0 (0)
	Non-cons	73	0.0 (0)	4.1 (3)	8.2 (6)	31.5 (23)	56.2 (41)	0.0 (0)
	IF experience	69	1.4 (1)	4.3 (3)	8.7 (6)	36.2 (25)	49.3 (34)	0.0 (0)
	No IF experience	79	0.0 (0)	1.3 (1)	6.3 (5)	36.7 (29)	55.7 (44)	0.0 (0)
	Total	148	0.7 (1)	2.7 (4)	7.4 (11)	36.5 (54)	52.7 (78)	0.0 (0)
End of bed clinical impression	Cons	75	2.7 (2)	0.0 (0)	12.0 (9)	29.3 (22)	56.0 (42)	0.0 (0)
	Non-cons	73	1.4 (1)	4.1 (3)	11.0 (8)	39.7 (29)	43.8 (32)	0.0 (0)
	IF experience	69	0.0 (0)	1.4 (1)	10.1 (7)	34.8 (24)	53.6 (37)	0.0 (0)
	No IF experience	79	3.8 (3)	2.5 (2)	12.7 (10)	34.2 (27)	46.8 (37)	0.0 (0)
	Total	148	2.0 (3)	2.0 (3)	11.5 (17)	34.5 (51)	50.0 (74)	0.0 (0)

Responses as % (*n*). Note ST7-8 category also includes post-CCT fellows.

CCT = certificate of completion of training; cons = consultant; IF = intestinal failure; IFU = intestinal failure unit; MUST = Malnutrition Universal Screening Tool; non-cons = nonconsultant; SAS = specialty and associate specialist; ST = specialty trainee; WAASP = weight, appetite, ability to eat, stress factor, pressure ulcer/wound.

There was wide variation in the use of MUST (Malnutrition Universal Screening Tool), body mass index and percentage weightloss over time, with consultants using each method significantly more than nonconsultants (Table 3). Nonconsultants used albumin (65.7%, 48/73) more than consultants (60.0%, 45/75). Except for the use of the MUST score, or its Wales equivalent (weight, appetite, ability to eat, stress factor, pressure ulcer/wound), IF experience did not confer any significant difference to which tool or method was used to identify malnutrition (Supplementary Table S1).

### Nutrition training and the future

A majority of surgeons (81.7%, 121/148), across all grades and IF experience, responded that the training they received does not allow them to identify malnourished surgical patients (Table 4). Despite this, consultants and those with IF experience reported a far greater likelihood of using evidence-based medicine when determining whether a patient is malnourished compared with nonconsultants and surgeons with no IF experience.

**Table 4:** Training, use of evidence-based practice and collaboration with colleagues according to surgeon seniority and experience of intestinal failure

Question	Role	*n*	Never	Rarely	Occasionally	Regularly	All the time	I do not know
The training which surgeons receive allows them to identify all malnourished emergency surgical patients	Cons	75	6.7 (5)	45.3 (34)	29.3 (22)	16.0 (12)	1.3 (1)	1.3 (1)
	Non-cons	73	9.6 (7)	53.4 (39)	19.2 (14)	16.4 (12)	1.4 (1)	0.0 (0)
	IF experience	69	4.3 (3)	47.8 (33)	29.0 (20)	15.9 (11)	2.9 (2)	0.0 (0)
	No IF experience	79	11.4 (9)	50.6 (40)	20.3 (16)	16.5 (13)	1.3 (1)	0.0 (0)
	Total	148	8.1 (12)	49.3 (73)	24.3 (36)	16.2 (24)	1.4 (2)	0.7 (1)
I use evidence-based practice when deciding a patient is malnourished	Cons	75	1.3 (1)	16.0 (12)	29.3 (22)	42.7 (32)	6.7 (5)	4.0 (3)
	Non-cons	73	4.1 (3)	19.2 (14)	37.0 (27)	30.1 (22)	2.7 (2)	6.8 (5)
	IF experience	69	0.0 (0)	17.4 (12)	26.1 (18)	47.8 (33)	5.8 (4)	2.9 (2)
	No IF experience	79	5.1 (4)	17.7 (14)	39.2 (31)	26.6 (21)	3.8 (3)	7.6 (6)
	Total	148	2.7 (4)	17.6 (26)	33.1 (49)	36.5 (54)	4.7 (7)	5.4 (8)
I seek help or advice from colleagues when deciding a patient is malnourished	Cons	75	1.3 (1)	14.7 (11)	18.7 (14)	40.0 (30)	25.3 (19)	0.0 (0)
	Non-cons	73	0.0 (0)	6.8 (5)	21.9 (16)	49.3 (36)	21.9 (16)	0.0 (0)
	IF experience	69	0.0 (0)	14.5 (10)	17.4 (12)	42.0 (29)	26.1 (18)	0.0 (0)
	No IF experience	79	1.3 (1)	7.6 (6)	22.8 (18)	46.8 (37)	21.5 (17)	0.0 (0)
	Total	148	0.7 (1)	10.8 (16)	20.3 (30)	44.6 (66)	23.6 (35)	0.0 (0)

Responses as % (*n*). Note ST7-8 category also includes post-CCT fellows.

CCT = certificate of completion of training; cons = consultant; IF = intestinal failure; IFU = intestinal failure unit; non-cons = nonconsultant; SAS = specialty and associate specialist; ST = specialty trainee.

Surgeons regularly seek advice from colleagues when deciding whether a patient is malnourished (Table 4). In order of frequency, advice from the nutrition support team, learning from previous surgical cases and advice from other surgical colleagues are the main approaches surgeons used to do so (Supplementary Table S6). This is regardless of surgeon seniority or IF experience (Supplementary Table S6). Undergraduate training is lacking, with only one in eight surgeons reportedly being trained at medical school; even fewer (6.6%, 25/379 responses) reported to have attended a postgraduate training course.

For all participants, regardless of surgeon seniority or IF experience, a specific local teaching session or a short course aimed at surgeons in training were reported to be most helpful in improving nutritional assessment/management. Only one in five recommend additional teaching at medical school (Table 5).

**Table 5:** Methods to improve nutrition training according to surgeon seniority and experience of intestinal failure

Role	*n*	Specific teaching at medical school	A specific local or Deanery teaching session	A 1-2 day course aimed at surgeons in training	A postgraduate course	I do not know	Other
Cons	135	20.0 (27)	26.7 (36)	34.1 (46)	14.8 (20)	1.5 (2)	3.0 (4)
Non-cons	114	17.5 (20)	36.0 (41)	28.9 (33)	14.9 (17)	2.6 (3)	0.0 (0)
IF experience	123	17.9 (22)	30.9 (38)	33.3 (41)	14.6 (18)	1.6 (2)	1.6 (2)
No IF experience	126	19.8 (25)	31.0 (39)	30.2 (38)	15.1 (19)	2.4 (3)	1.6 (2)
Total	249	18.9 (47)	30.9 (77)	31.7 (79)	14.9 (37)	2.0 (5)	1.6 (4)

Responses as % (*n*). Note ST7-8 category also includes post-CCT fellows.

CCT = certificate of completion of training; cons = consultant; IF = intestinal failure; IFU = intestinal failure unit; non-cons = nonconsultant; SAS = specialty and associate specialist; ST = specialty trainee.

### Free text comments

Surgeons offered many comments regarding aspects of nutrition practice and training, which reiterated the variability in practice and methods sought to learn about surgical nutrition (Supplementary Table S7).

## Discussion

There is wide variation in practice among consultant and nonconsultant general surgeons. Regardless of surgeon seniority or experience of IF, malnutrition is considered an important component of being a general surgeon, but it is poorly taught at undergraduate and postgraduate level.

### Perceptions around malnutrition in EGS

Almost all surgeons appreciated that a patient's nutritional status impacts surgical outcomes, and identification of malnutrition was recognised as an important skill. This was irrespective of seniority or experience of IF. In contrast, only two-thirds of surgeons reported regularly screening for malnutrition, or felt it was their role to do so from the outset. A similar study reports participants screened only 25% of patients every time.^6^ Even fewer surgeons felt it was their role to manage malnourished EGS patients. There were no significant differences according to seniority or IF experience. Together, surgeons considered these processes should be led by dietitians and the nutrition support team. This lack of ownership has been documented previously.^5,20^

These findings may be explained by a general lack of confidence in identifying and managing malnourished EGS patients due to a lack of specific nutrition training as shown in this study. The knowledge and skills gained from working with IF patients likely translate into more confident and routine practice of identifying the potential for such patients. There are no statutory assessments for this skill specifically in the trainee surgical curriculum.^21^ Clearly, adequate training will boost confidence and may empower surgeons to feel involved in decision making around nutritional support for patients.

### Current practices in identifying malnutrition

As previously reported, several methods to identify malnourished patients prevail.^5,22^ NICE suggest the Malnutrition Universal Screening Tool (MUST) is an acceptable tool to screen for malnutrition, and it is widely adopted in the UK. Forty percent of surgeons in this study report using it, or the Wales equivalent WAASP, regularly or all the time. This is not too dissimilar from our previous study whereby over half of NELA Leads reported MUST was the screening tool of choice compared with other tools.^5^ However, of all screening methods presented, it was reported to be the least used method by which surgeons identify malnutrition. This may reflect its inability to stratify malnutrition risk in the EGS cohort, where many such patients may be high risk on MUST but not in need of nutritional intervention beyond dietetic involvement or monitoring. It may lead to the perception by surgeons that screening for malnutrition is unlikely to change the immediate management, the commonest reason cited for not doing so in this study.

On the contrary, more than 95% of surgeons used ‘reduced oral intake until now’, and almost 90% used ‘reduced oral intake from now’. A national study of UK patients with small bowel obstruction showed more than half who were considered to be at severe risk of malnutrition had no enteral intake for more than five days, with a 16% in-hospital mortality rate.^1^ A patient's oral intake relies on a thorough history and is ideally obtained at the start of their admission. Predicting future oral intake is subject to several dynamic elements of a patient's journey through hospital. However, it is paramount to recognise the additive effect of few days of ‘reduced oral intake until now’ as well as a few days of ‘reduced oral intake from now’. Patients may have little to no oral intake for longer than we realise. They may become malnourished during their admission and have a significant risk of dying in hospital. The story begins from the onset of symptoms, not from their first presentation to hospital.

Albumin was reported to be used commonly by surgeons to identify malnourished patients. It is used less frequently by more senior surgeons and those with IF experience, which may confer a familiarity with guidelines recommending not using it.^9,12,23–25^ We have previously explored the use of albumin in the acute setting,^5^ and reiterate that although it may indicate potential disastrous surgical outcomes, it should not be used specifically to identify malnourished patients.

### Nutrition training and the future

At the end of training, surgeons are expected to ‘recognise and assess nutritional requirements of the patient and appropriate routes of administration of nutrition’.^21^ However, surgeons reported a worrying lack of nutrition training in the UK at undergraduate and postgraduate level. Other studies of undergraduates, foundation doctors^26^ and surgeons^7^ report similar findings. Whereas foundation doctors report training consisting of two hours in the past year,^26^ almost all surgeons report never having received any training in nutrition.^7^ Typically, there is no formal nutrition-based education for trainee surgeons beyond scattered clinical experiences. This may explain why less than half of surgeons including consultants felt they had the necessary skillset to identify malnourished surgical patients.

The variability of nutrition-related learning outcomes across a range of postgraduate programmes has been documented.^27^ Although the gastroenterology curriculum substantially leads general surgery for the number of nutrition-related outcomes a part of training, both far surpass other specialties. It is worth noting that gastroenterologists play a vital role in the nutrition support teams from which surgeons regularly seek advice. However, similar to the findings of this study, trainee gastroenterologists recognise the importance of nutrition but have low confidence in managing IF as a result of inadequate training opportunities.^8^ This is despite an expectation of 3–6 months nutrition-focused training, with some trainees undertaking a year-long ‘advanced training placement’ at a specialist centre, neither of which surgeons undertake. Mandating surgical trainees to undertake such rotations, although aspirational, will be difficult to achieve in an already compressed programme. However, the sheer volume of malnourished surgical EGS patients and deleterious outcomes necessitates urgent change. The management of malnourished EGS patients will not be reserved for specialised intestinal units.

A variety of strategies to address the lack of nutrition training are available. There has been much work on incorporating nutrition training in undergraduate medical education.^28,29^ From a surgical perspective, the involvement of surgeons in nutrition multidisciplinary teams, particularly for EGS patients, would be helpful. The vast majority of surgeons wanted specific local teaching or a short nutrition course aimed at surgeons in training. Findings from this study warrant the development of a formal nutrition-based educational intervention. The development of a postgraduate course may be of benefit, akin to the development of the nontechnical skills for surgeons (NOTSS),^30^ although performance change is greater with longer programmes rather than day-long short courses.^31^

### Strengths and limitations

This study is wide ranging and captures surgeon perceptions from every region across the UK, benefiting from research support from the ASGBI. Approximately equal numbers of consultants to nonconsultants, and surgeons with and without experience of IF is testament to this. Exploring the association of these factors with participant responses adds to the depth of this study.

The limitations of this study, however, are its relatively small numbers in comparison with registered general surgeons in the UK.^18^ Survey methodology may not be the most appropriate method to assess knowledge of surgical nutrition, and given it is considered by some surgeons to be a ‘nonsurgical’ component of surgical management,^32^ only those with an interest in nutrition may have been inclined to participate in this study. However, the study design included a pilot phase and a broad recruitment strategy with support from the ASGBI, both hopefully mitigating some of this bias. Further, experience of IF was not defined, although doing so may have been arbitrary in a competency-based curriculum and, again, the self-reported nature of survey methodology.

### Implications for policy makers and researchers

Although nutritional tools to identify malnourished patients in EGS exist,^22^ a knowledge gap prevails. Professional associations should consider developing a specific course to improve knowledge and understanding around nutrition in EGS. Primarily, it should promote early recognition and referral of patients that may become malnourished for a full nutritional assessment and consideration of nutritional support. Secondly, nutritional support pathways may contribute to this aspect in the clinical environment and could outline the roles of each team member explicitly. Understanding the key drivers of decision making around who should receive nutritional support is warranted and may guide development of such pathways.

## Conclusion

Regardless of seniority and IF experience, surgeons recognise malnutrition is important and can affect patient outcomes. They lack confidence in identifying malnourished EGS patients, and there is a desire for training to bridge the knowledge gap that exists.

## Data Availability

Data can be made available on request.
